# Impact of dialysis on intravesical recurrence and survival outcomes in upper tract urothelial cancer patients undergoing radical nephroureterectomy

**DOI:** 10.1080/0886022X.2025.2458762

**Published:** 2025-02-07

**Authors:** Chi-Chun Hsieh, Ching-Chia Li, Yung-Shun Juan, Wei-Ming Li, Wen-Jeng Wu, Tsu-Ming Chien

**Affiliations:** a Department of Urology, Kaohsiung Medical University Hospital, Kaohsiung, Taiwan; b Department of Urology, Kaohsiung Medical University Gangshan Hospital, Kaohsiung, Taiwan; c Department of Urology, School of Postbaccalaureate Medicine, College of Medicine, Kaohsiung Medical University, Kaohsiung, Taiwan

**Keywords:** Upper tract urothelial carcinoma, dialysis, radical nephroureterectomy, intravesical recurrence, end-stage renal diseases

## Abstract

**Background:**

Upper tract urothelial carcinoma (UTUC) presents a significant recurrence risk following radical nephroureterectomy (RNU). Patients on dialysis may experience unique clinical trajectories due to uremic states and altered immune responses.

**Objective:**

To evaluate the impact of dialysis on intravesical recurrence and survival outcomes in patients with UTUC undergoing RNU, and to identify predictive factors influencing prognosis.

**Methods:**

A retrospective cohort study analyzed 402 patients with non-metastatic UTUC treated with RNU between 2001 and 2014. Patients were stratified into dialysis (*n* = 66) and non-dialysis (*n* = 336) groups. Survival and recurrence outcomes were assessed using Kaplan–Meier and Cox regression analyses.

**Results:**

Dialysis patients were predominantly female, younger, and exhibited less advanced pathological tumor stages. Dialysis was associated with higher intravesical recurrence rates (*p* = 0.009), which were largely attributable to a history of bladder cancer (42.4% vs. 26.5%; *p* = 0.009). After adjustment for bladder cancer history, dialysis was not an independent predictor of bladder recurrence-free survival (BRFS). Advanced pT stages (HR: 3.9, *p* = 0.012) and prior bladder cancer were the primary factors influencing BRFS.

**Conclusions:**

Dialysis does not independently worsen surgical outcomes or BRFS in UTUC patients post-RNU when accounting for prior bladder cancer. Prognostic models should integrate these findings to enhance individualized surveillance and treatment strategies.

## Introduction

Patients with end-stage renal disease (ESRD) receiving dialysis have been reported to be at increased risk for various cancers [1–3]. An international collaborative study has shown that the excess risk can mainly be ascribed to the effects of underlying urologic diseases and increased susceptibility to viral carcinogenesis [3]. One population-based study in the US showed that the risk was most elevated for cancers of the urinary tract. Another population-based cohort study in Taiwan showed a trend of increased risk of cancer in young ESRD patients, and within the first year of dialysis [2]. These studies suggested that it is necessary to develop specific strategies for different ethnicities for cancer screening of chronic dialysis patients. Another study showed that patients with ESRD on hemodialysis had a high risk of developing urothelial carcinoma (UC), especially UC of the upper urinary tract (UTUC), in Taiwan [4]. This high burden of cancer risk in the dialysis population is viewed as an important public health issue, and more frequent urine cytology and upper urinary tract surveys are needed in hemodialysis patients.

At present, the overall and age-standardized global incidence of ESRD requiring dialysis is increasing worldwide; however, the trend has been stable or even decreased in some developed countries [5, 6]. On the other hand, Taiwan demonstrated the highest ranking of incidence of dialysis worldwide in the annual report of the United States Renal Database System (USRDS) in 2023 [7], and the annual incidence and prevalence of dialysis has been steadily increasing in Taiwan [8].

Urothelial carcinoma, which includes UC of the urinary bladder (UBUC) and UTUC, is the most common malignancy of the urinary tract. Increasing evidence suggests that UTUC should be considered a different disease entity from UBUC, even though both are urothelial in origin [9, 10]. For example, it has been reported that approximately 66% of UTUC patients have invasive disease at initial diagnosis, in comparison to 15–25% of patients presenting with muscle-invasive UBUC [11]. The ideal management of UTUC also differs from that of UBUC, ranging from surgical intervention, postoperative instillation therapy, and postoperative surveillance to medical management [9].

Although UTUC is reported to be uncommon in most Western countries, it has epidemic potential in some specific areas [12]. Raman et al. [13] reported an increase in the overall incidence of UTUC in the US, from 1.88 to 2.06 cases per 100,000 person-years, from 1973 to 2005. In addition, an abnormally high incidence of UTUC has been reported in some regions of the Balkans and Taiwan [12].

At present, the standard treatment for invasive, non-metastatic UTUC is radical nephroureterectomy (RNU) with bladder cuff excision. However, despite optimal treatment, a high risk of disease recurrence and progression are reported for UTUC, especially in the advanced stage [14]. Several prognostic factors for UTUC have been proposed in recent reports [14–18], which are mainly related to tumor characteristics and treatments. Clinical factors, such as pathological tumor stage and grade, are associated with divergent outcomes in patients with identical findings; therefore, they are insufficient for detailed risk stratification and the patient’s prognosis is difficult to define before treatment [18].

This high burden of cancer risk in the dialysis population is viewed as an important public health issue. Intravesical recurrence after RNU is a frequent event, ranging from 22.5 to 50% [18–24], requiring intense cystoscope surveillance. Several studies have tried to explore the risk factors of intravesical recurrence [19–24]; however, several heterogenous clinical characteristics, such as a history of bladder cancer or concomitant bladder cancer, previous intravesical therapy, and metastasis, were not clarified. In addition, there have been few studies that focused on the association of dialysis with UTUC patients after RNU [24, 25]. Wu et al. [25] concluded that patients with UTUC on dialysis have a high recurrence rate and metachronous or even multiple. Lin et al. [24] showed dialysis increases the risk of intravesical recurrence in patients with UTUC for unknown reasons. In the present study, the objective was to evaluate the impact of dialysis on patients with UTUC receiving RNU and to explore predictive factors for intravesical recurrence.

## Patients and methods

### Patients

Patients who underwent either open or laparoscopic RNU with bladder cuff excision for non-metastatic UTUC at Kaohsiung Medical University Hospital, Kaohsiung, Taiwan, between 2001 and 2014 were included in this study. The present study was approved by the review board of our institution (KMUH-IRB-20120138). Patients were divided into two groups according to their dialysis status before the operation. The dialysis group was defined as patients who had been diagnosed according to ICD-9-CM codes and procedure codes before UTUC management in our hospital. Clinical parameters, including demographic characteristics, pathological features, oncologic follow-up, and the cause leading to mortality, were retrospectively collected. Patients with neoadjuvant chemotherapy or radiotherapy, concurrent bladder tumor, acute blood disorders, or incomplete clinical information were excluded. In order to clarify the impact of dialysis on UTUC patients, we also excluded those patients newly undergoing dialysis after the surgery. Tumor stage was evaluated according to the 2002 American Joint Committee Cancer TNM system. All cases were reviewed by two pathologists and re-classified as low or high grade using the 2004 World Health Organization grading system.

### Postoperative follow-up

After the operation, outpatient clinic visits were arranged every 3 months in the first 2 years and every 6 months in the subsequent 2 years. From the fifth year onwards, annual follow-ups were arranged for patients with no evidence of disease. Detailed history-taking, physical examination, urine cytology, cystoscopy, and serial imaging survey were performed, following the surveillance guidelines. Tumor recurrence was defined as local recurrence at the operative site, in regional lymph nodes, or a distant metastasis. Tumors occurring in the bladder or contralateral upper urinary tract were considered metachronous and were not categorized as disease recurrence. Patients who were treated with bladder tumor excision or cystectomy over three months after RNU were identified as having bladder recurrence. Adjuvant chemotherapy and radiation therapy were administered to 82 and 35 patients, respectively, according to pathological stage, performance status, renal function, and consent to treatment.

### Statistical analysis

Differences between categorical parameters were assessed using the χ2 or Fisher’s exact test. The Kaplan–Meier method was applied to estimate the effects of dialysis on overall survival (OS), cancer-specific survival (CSS), and bladder recurrence-free survival (BRFS). Survival rates were recorded from the day of RNU to death, cancer-specific death, bladder recurrence, or the latest visit. Survival curves were compared using a log-rank test. The model for predicting bladder recurrence was developed based on univariate analysis, with only factors significant at *p* < 0.05 included in the multivariate Cox proportional hazards model to identify clinical and pathologic variables significantly associated with recurrence and survival rates. To assess the Cox proportional hazard assumption, we tested the proportionality of hazards over time by adding interactions between time and covariates into the model. None of the interactions were significant, indicating that the proportional hazards assumption was not violated. Statistical significance was set at *p* < 0.05. SPSS 20.0 (SPSS Inc., Chicago, IL, USA) was used for all statistical analyses. Due to the retrospective nature of the study, if there are any missing data in the data collection, we used multiple imputation in SPSS to fill in the gaps.

## Results

There were 482 patients who underwent RNU, and 38 patients (7.8%) had concurrent non-muscle invasive UBUC, 5 patients (1.0%) had concurrent muscle invasive UBUC, 3 patients (0.6%) had contralateral UTUC, 1 patient (0.2%) had paraneoplastic leukocytosis, and 24 patients (5%) progressed to ESRD requiring permanent dialysis after surgery. Overall, from among these patients, we included 402 patients(dialysis, 66 (16.4%) patients; non-dialysis 336 (83.6%) patients) in the current study.

Preoperative uretereroenoscopic (URS) examination and biopsy were performed in 338 patients (84.0%), image-guided biopsy in 48 patients (11.9%), and 16 patients (4%) presented suspicious imaging features. There were 66 patients (16.4%) undergoing dialysis before RNU. Table 1 shows the patients’ clinical and pathologic profiles. The mean ± standard deviation in the age of patients who underwent RNU was 66.9 ± 10.5 years (62.4 ± 10.7 years in the dialysis group and 67.7 ± 10.3 years in the non-dialysis group, *p* < 0.001). The mean follow-up time after surgery was 39.2 ± 31.5 months (37.8 ± 30.9 months in the dialysis group and 39.4 ± 31.7 months in the non-dialysis group). No difference in follow-up time was observed between the two groups (Table 2).

**Table 1.** Demographics and clinicopathologic characteristics of 402 patients with UTUC according to hemodialysis or not.

		Hemodialysis		
	Total	Yes	No	
Variables	*n* = 402	*n* = 66	*n* = 336	*p* value
Age (Years)				
Mean ± SD	66.9 ± 10.5	62.4 ± 10.7	67.7 ± 10.3	**<0.001**
Over 65 years	245 (60.9)	27 (40.9)	218 (64.9)	**<0.001**
Smoking				**0.028**
Yes	83 (20.6)	7 (10.6)	76 (22.6)	
Male Gender				**<0.001**
Yes	162 (40.3)	12 (18.2)	150 (44.6)	
BMI (kg/m^2^)				
Mean ± SD	23.6 ± 3.9	23.9 ± 3.7	23.6 ± 4.2	0.754
ECOG score				0.174
0, 1	358 (89.1)	61 (92.4)	293 (87.2)	
2, 3	44 (10.9)	5 (7.6)	43 (12.8)	
Preoperative hydronephrosis				**0.005**
Yes	68 (16.9)	19 (28.8)	49 (14.6)	
Bladder cancer history				**0.009**
Yes	117 (29.1)	28 (42.4)	89 (26.5)	
Type of operation				**0.007**
Open	239 (59.5)	49 (74.2)	190 (56.5)	
Laparoscopic	163 (39.9)	17 (25.8)	146 (43.5)	
Tumor location				**0.007**
Pyelocaliceal	154 (38.3)	20 (30.3)	134 (39.9)	
Ureteral	175 (43.5)	25 (37.9)	150 (44.6)	
Both	73 (18.2)	21 (31.8)	52 (15.5)	
Multifocality				**<0.001**
Single	322 (80.1)	37 (56.1)	285 (84.8)	
Multifocal	80 (19.9)	29 (43.9)	51 (15.2)	
Pathological tumor stage				0.180
pTis/pTa/pT1	164 (40.8)	32 (48.5)	132 (39.3)	
pT2	100 (24.9)	20 (30.3)	80(23.8)	
pT3	113 (28.1)	11 (16.7)	102 (30.4)	
pT4	25 (6.2)	3 (4.5)	22 (6.5)	
Advanced pathological tumor stage (pT3/pT4)				
Yes	138 (34.3)	14 (21.2)	124 (36.9)	**0.014**
Lymph node invasion				
Yes	79 (19.7)	13 (19.7)	66 (19.6)	0.750
Grade				0.104
High	311 (77.4)	46 (69.7)	265 (78.9)	
Low	91 (22.6)	20 (30.3)	71 (21.1)	
Adjuvant chemotherapy				**<0.001**
Yes	82 (20.4)	2 (3.0)	80 (23.8)	
Radiation therapy				0.074
Yes	35 (8.7)	2 (3.0)	33 (9.8)	

BMI: body mass index; CKD: Chronic kidney disease; ECOG: Eastern Cooperative Oncology Group; UTUC: upper tract urothelial carcinoma.

Bold values are statistically significant at *p* < 0.05.

**Table 2.** Univariate and multivariate analyses predicting OS, CSS and BRFS in patients (*n* = 402) with UTUC after RNU.

	OS				CSS				BRFS			
	Univariate Analysis		Multivariate analysis		Univariate analysis		Multivariate analysis		Univariate analysis		Multivariate analysis	
Parameters	HR (95%CI)	*p*	HR (95%CI)	*p*	HR (95%CI)	*p*	HR (95%CI)	*p*	HR (95%CI)	*p*	HR (95%CI)	*p*
Age (Years)												
Over 65 years	1.3 (0.9–1.7)	0.098			1.0 (0.8–1.2)	0.969			1.0 (0.8–1.4)	0.836		
Smoking												
Yes vs No	1.6 (1.1–2.8)	**0.042**	1.9 (1.1–3.5)	**0.046**	1.1 (0.7–1.7)	0.764			1.0 (0.7–1.6)	0.839		
BMI (Kg/m^2^)												
Over 27 Kg/m^2^	1.1 (0.9–1.4)	0.217			1.2 (0.9–1.4)	0.109			1.1 (0.8–1.3)	0.913		
ECOG												
2, 3 vs 0, 1	1.5 (0.8–2.8)	0.241			1.4 (0.9–2.4)	0.225			1.2(0.8–2.0)	0.538		
CKD stage												
Stage 2 vs Stage 0,1	1.1 (0.4–3.4)	0.900			2.9 (0.4–4.1)	0.255			1.2 (0.5–3.6)	0.520		
Stage 3 vs Stage 0,1	2.4 (0.8–7.4)	0.104			3.1 (0.5–4.7)	0.367			1.3 (0.6–3.4)	0.442		
Stage 4 vs Stage 0,1	1.6 (0.4–7.1)	0.635			3.9 (0.7–4.7)	0.283			1.3 (0.3–3.9)	0.823		
Stage 5 vs Stage 0,1	1.3 (0.4–3.6)	0.681			3.1 (0.6–4.7)	0.360			1.7 (0.7–4.3)	0.214		
Advanced CKD (stage 4, 5)												
Yes vs No	1.2 (0.8–1.7)	0.438			1.8 (1.1–2.8)	**0.008**	1.2 (0.5–2.7)	0.686	1.1 (0.9–1.2)	0.466		
Bladder cancer history												
Yes vs No	1.1 (0.9–1.3)	0.136			1.1 (0.9–1.3)	0.136			2.8 (2.1–3.7)	**<0.001**	4.4 (2.3–12.2)	**<0.001**
Dialysis												
Yes vs No	1.0 (0.9–1.1)	0.935			2.5 (1.2–4.9)	**0.007**	1.8 (0.7–5.1)	0.244	1.6 (1.2–2.8)	**0.040**	1.2 (0.6–2.4)	0.655
Preoperative hydronephrosis												
Yes vs No	1.0 (0.9–1.1)	0.603			1.1 (0.71.8)	0.701			1.2 (1.0–1.3)	**0.026**	2.3 (1.1–4.8)	**0.030**
Type of operation												
Laparoscopic vs open	0.6 (0.3–1.2)	0.125			0.9 (0.6–1.5)	0.803			0.7 (0.4–1.1)	0.158		
Tumor location												
Ureteral vs Pyelocaliceal	1.4 (1.2–2.6)	**0.041**	1.3 (0.9–1.7)	0.129	1.3 (0.8–2.1)	0.257			1.0 (0.6–1.7)	0.967		
Both vs Ureteral	1.2 (0.6–2.5)	0.683			1.5 (0.5–4.3)	0.408	1.2 (0.6–2.6)	0.211	1.4 (0.8–2.7)	0.238		
Both vs Pyelocaliceal	1.4 (0.7–2.9)	0.342			1.6 (0.4–4.2)	0.320	2.7 (0.8–9.0)	0.195	1.5 (0.8–2.6)	0.204		
Pathologic T stage												
pT2 vs pTa/pTis/pT1	1.7 (0.6–4.8)	0.289	1.4 (0.4–4.1)	0.593	2.8 (1.2–7.0)	**0.008**	2.2 (0.2–3.8)	0.195	1.1 (0.6–1.8)	0.914	1.0 (0.6–2.2)	0.683
pT3 vs pTa/pTis/pT1	7.2 (3.3–16.6)	**<0.001**	2.3 (1.5–3.5)	**<0.001**	11.2 (6.2–22.7)	**<0.001**	3.8 (0.8–4.5)	0.187	1.2 (0.7–1.9)	0.823	1.2 (0.9–2.1)	0.113
pT4 vs pTa/pTis/pT1	9.7 (3.8–27.4)	**<0.001**	2.6 (1.3–5.4)	**<0.001**	12.6 (5.2–35.3)	**<0.001**	6.6 (4.5–8.7)	**<0.001**	3.9 (1.1–6.4)	**0.010**	2.1 (1.1–3.2)	**0.042**
Advanced T stage												
Yes vs No	6.5 (3.3–11.4)	**<0.001**	3.0 (1.6–5.3)	**<0.001**	1.2 (1.1–1.3)	**<0.001**	1.9 (0.8–2.9)	0.387	1.8 (1.2–2.6)	**0.030**	3.9 (1.3–11.1)	**0.012**
Multifocality												
Multifocal vs Single	1.5 (0.8–2.8)	0.217			2.2 (1.2–3.8)	**<0.001**	1.6 (0.7–3.9)	0.385	1.1 (0.6–1.9)	0.307		
Lymph node invasion												
Yes vs No	2.6 (1.6–4.3)	**<0.001**	1.8(1.6–3.5)	**<0.001**	3.8 (2.2–6.4)	**<0.001**	1.2 (0.5–2.9)	0.640	2.1 (0.8–5.3)	0.110		
Grade												
High vs Low	1.9 (1.1–3.1)	**0.011**	1.1 (0.4–3.6)	0.829	4.1 (2.0–8.5)	**<0.001**	2.3 (0.8–6.1)	0.108	1.0 (0.9–1.2)	0.434		
Adjuvant chemotherapy												
Yes vs No	8.9 (4.8–16.4)	**<0.001**	6.6 (3.2–13.7)	**<0.001**	2.0 (1.6–2.5)	**<0.001**	8.0 (4.2–15.2)	**<0.001**	1.3 (1.1–2.4)	**<0.001**	5.1 (2.5–10.5)	**<0.001**
Anemia												
Yes vs No	1.1 (0.7–1.8)	0.995			1.1 (0.7–1.5)	0.699			1.2 (0.9–1.8)	0.132		

BMI: body mass index; CKD: chronic kidney disease; ECOG: Eastern Cooperative Oncology Group; UTUC: upper tract urothelial carcinoma.

Bold values are statistically significant at *p* < 0.05.

Dialysis patients were associated with younger age, predominantly female, symptomatic hydronephrosis, bladder cancer history, open operation method, multiple tumors, multifocal tumors, less advanced pathological tumor stage, and less adjuvant chemotherapy than those without dialysis (Table 1).

### Kaplan–Meier analysis for OS, CSS, and BRFS

**Figure 1.**
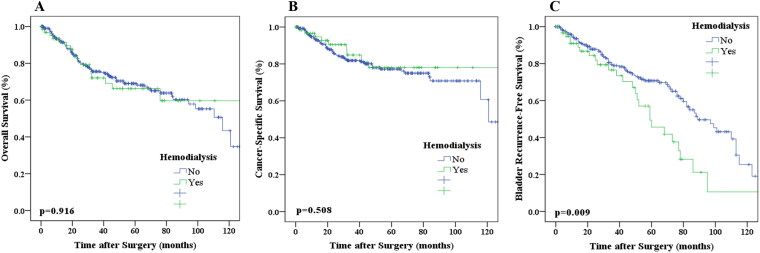
Kaplan–Meier estimates of survival in patients with UTUC based on hemodialysis. A, OS. B, CSS. C, BRFS.

A total of 102 patients (25.4%) from our study cohort experienced death events (dialysis, 17 patients; non-dialysis, 85 patients). The 3- and 5-year OS rates were 75.5% and 68.1%, respectively. Kaplan–Meier analysis indicated that the OS rates were not significantly correlated with dialysis status (*p* = 0.916; Figure 1(A)). Univariate analysis showed that smoking (*p* = 0.042), ureteral tumor location (*p* = 0.041), advanced T-stage tumor (*p* < 0.001), lymph node invasion (*p* < 0.001), high-grade tumor (*p* = 0.011), and adjuvant chemotherapy (*p* < 0.001) were associated with worse OS. Meanwhile, smoking (*p* = 0.046), advanced T stage, lymph node invasion (*p* < 0.001), and adjuvant chemotherapy (*p* < 0.001) were independent risk factors for poor OS.

A total of 68 patients (16.9%) underwent cancer-specific death events (dialysis, 9 patients; non-dialysis, 59 patients). The 3- and 5-year CSS rates were 78.8% and 75.8%, respectively. Kaplan–Meier analysis proved that the CSS rates were not related to dialysis status (*p* = 0.370; Figure 1(B)). Univariate analysis showed advanced CKD stages (*p* = 0.008; defined as CKD in stage 4 or 5 in this study), dialysis status (*p* = 0.007), advanced T stage (*p* < 0.001), multifocal tumor (*p* < 0.001), lymph node invasion (*p* < 0.001), high-grade tumor (*p* < 0.001), and adjuvant chemotherapy (*p* < 0.001) were associated with worse CSS. Meanwhile, pT4 tumor (*p* < 0.001) and adjuvant chemotherapy (*p* < 0.001) were independent risk factors for a shorter CSS in the multivariate analysis.

A total of 110 patients (27.3%) had intravesical recurrence (dialysis, 27 patients; non-dialysis 83 patients). The 3- and 5-year BRFS rates were 78.9% and 70.7%, respectively. Kaplan–Meier analysis revealed that the BRFS rates were significantly related to dialysis status (*p* = 0.009; Figure 1(C)). Univariate analysis showed that bladder cancer history (*p* < 0.001), dialysis status (*p* = 0.04), symptomatic hydronephrosis (*p* = 0.026), pT4 tumor (*p* = 0.01), advanced T stage (*p* = 0.03), and adjuvant chemotherapy (*p* < 0.001) were related to worse BRFS. Meanwhile, the multivariate analysis found that bladder cancer history (*p* < 0.001), pT4 tumor (*p* = 0.042), advanced pT stages (*p* = 0.012), and adjuvant chemotherapy (*p* < 0.001) were associated with poor BRFS rates.

### Dialysis and BRFS

**Figure 2.**
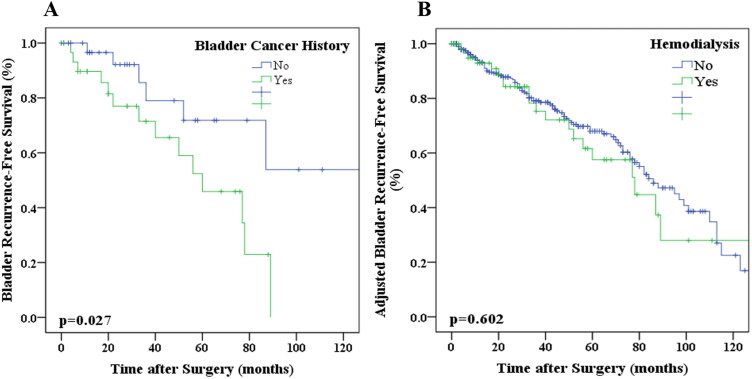
Kaplan–Meier estimates of survival in patients with UTUC based on bladder cancer history. A, BRFS. Kaplan–Meier estimates of survival in patients with UTUC based on hemodialysis. B, adjusted BRFS.

**Figure 3.**
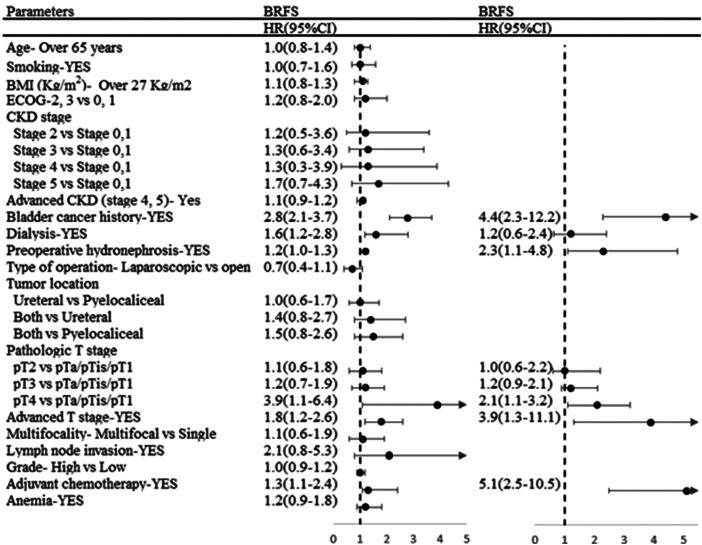
Crude and adjusted hazard ratios for BRFS.

Univariate analysis revealed bladder cancer history and dialysis as risk factors for increased bladder recurrence. Dialysis failed to reach statistical significance as an independent factor in the multivariate analysis. Kaplan–Meier analysis showed that bladder cancer history was significantly associated with BRFS (*p* = 0.027; Figure 2(A)). After adjusting for bladder cancer history, no significant correlation was found between dialysis and BRFS (*p* = 0.602; Figure 2(B)). Crude and adjusted hazard ratios for BRFS were shown in Figure 3.

## Discussion

In the present study, 27.3% of all patients experienced intravesical recurrence. The bladder recurrence rate among patients with a history of previous bladder cancer was 41.9%. Meanwhile, the intravesical recurrence rate after RNU without a history of bladder cancer was 21.4%. These results are consistent with those of previous studies [18–24]. We believe that the causes of bladder recurrence differ between the patients with and without a history of bladder cancer. For non-muscle-invasive bladder cancer, undetected cancer upon cystoscopy, local residual disease after transurethral resection, and tumor re-implantation might cause early disease recurrence, and field change cancerization effects could lead to late disease recurrence [26]. Cell dissemination within the lumen followed by cell grafting to the urinary tract wall during both natural and perioperative processes are believed to the main causes of intravesical recurrence in UTUC patients having undergone RNU [23, 26]. However, not all seeding cancer cells from the UTUC will graft to the bladder wall, while a previous report has shown that multifocal and high-grade UTUC tumors have such intravesical implantation potential [23]. Our study revealed that advanced tumor stage had the same implantation potential.

The EAU guidelines have demonstrated that a single post-operative intravesical instillation therapy soon after surgery reduces the risk of bladder recurrence within the initial years [16]. We also previously proved the efficacy of prophylactic intravesical chemotherapy for UTUC after RNU, and showed that the instillation of epirubicin or mitomycin C appears to be well tolerated and effective for preventing bladder recurrence and prolonging the time to first bladder recurrence [27]. We fully agree regarding the issue of the clinical benefits of single post-operative intravesical instillation therapy. Meanwhile, different approaches to the bladder cuff at the time of surgery remain unclear in terms of the prognostic significance. We previously compared the oncologic outcomes following RNU using three different methods for managing the bladder cuff: intravesical incision, extravesical incision, and transurethral incision (TUI) [28]. Our results showed no differences in bladder recurrence and cancer-specific survival among the three groups, but the TUI method seemed to have a lower—although not significantly different—recurrence rate; for example, regarding intravesical, extravesical, and TUI techniques, bladder recurrence developed in 23.5%, 24.0%, and 17.6% of cases (*p* = 0.485); local retroperitoneal recurrence in 7.4%, 7.8%, and 5.5% of cases (*p* = 0.798); contralateral recurrence in 4.9%, 3.9%, and 2.2% of cases (*p* = 0.632); and distant metastasis in 7.4%, 10.4%, and 5.5% of cases (*p* = 0.564), respectively. Therefore, we have preferentially used the TUI method in our hospital in recent years. As we do not suture the bladder cuff opening using the TUI method, the post-operative extravasation rate is much higher than those with bladder suturing. As a result, immediate intravesical instillation has not become a common practice in our hospital in recent years.

It has been suggested that dialysis patients have a reduced urine flow, with a high content of toxic waste products from the urinary tract to the bladder. The progressive exposure to toxins may explain the higher incidence of UTUC than UBUC in dialysis patients [4]. Our study of a series of patients diagnosed with UTUC during treatment with dialysis yielded some important observations. First, dialysis patients were younger and predominantly female. Lin et al. [2] showed that the relative risk of overall cancers in female patients with dialysis was higher than that in male patients. Younger dialysis patients also revealed a higher incidence of cancer development, consistent with our results. The reasons for younger dialysis patients having higher cancer rates are unclear. Heiland et al. [29] defined this age phenomenon as a situation in which stages of tumor promotion and progression occur faster in younger patients. In general, young patients have better functioning repair systems to remove the malignant cells from the body. The deterioration of the cancer defense system and inactivation of important tumor suppressor genes are observed in advanced age and in patients either with or without chronic kidney disease. In young dialysis patients, the cancer defense mechanism and tumor suppressor genes were impaired due to the uremic state. Therefore, the difference in cancer risk could disappear with advancing age.

In Taiwan, women use more traditional Chinese medicine treatments than men [30]. The national health insurance in Taiwan covers treatments involving herbal medicines, acupuncture, moxibustion, and other therapies. Sex differences were found to persist across the age groups. The regular consumption of herbal medicines is very common in Taiwan. Previous studies have reported a high national prevalence of UTUC in the country, which is associated with the use of carcinogenic remedies containing aristolochic acid [2, 4, 18, 24, 25, 27, 30]. Another interesting finding is that the incident dialysis cases predominantly included men in previous studies, whereas the prevalent dialysis cases were predominantly female before 2014 [8]. Hsiao et al. [4] found that females in a uremic cohort were prone to developing UTUC. While our current study also reached the same conclusion, the mechanism underlying this sex difference is still unclear.

Second, dialysis patients were in the early stages (Tis/Ta/T1, T2) at diagnosis compared to those with normal renal function. In our study group, over 90% of patients had the initial clinical symptom of painless gross hematuria, regardless of renal function. Oliguria or anuria was commonly seen in dialysis patients. Therefore, hematuria in this particular group should not be ignored, and may be considered as a symptom of a possible urological tumor until proven otherwise. With early detection, an aggressive surgical intervention may provide a better outcome.

A retrospective study revealed that patients undergoing dialysis have a higher risk of bladder recurrence, for unknown reasons [24]. Our survival analysis also showed that dialysis was associated with an increased bladder recurrence rate. We noted that the incidence of previous bladder cancer was high in the dialysis group (42.4% vs. 26.5%, *p* = 0.009). A previous bladder cancer history has been shown to be one of the most important factors for bladder recurrence after RNU [15–17]. After adjusting for bladder cancer history, we showed that dialysis does not increase the risk of bladder recurrence. A history of bladder cancer indicates that a tumor originated in the bladder. Therefore, the origin of intravesical recurrence after RNU was not the same when compared to patients without a previous bladder tumor. We found the same results for the population that started dialysis after surgery; if we do not adjust for those with a history of bladder cancer, we find that the postoperative dialysis group increases the risk of bladder recurrence. However, after adjusting for this important factor, neither preoperative nor postoperative dialysis patients increase the risk of bladder recurrence after surgery. Moreover, many previous studies have included some very high-risk patients, who may have died due to metastatic disease before bladder recurrence. Careful patient selection and proper bias exclusion are crucial when identifying the risk factors for bladder recurrence after RNU.

This study had several limitations. First, this was a retrospective analysis of a single-center series. Second, the enrolled patients were treated by different surgeons over a 13-year period. Third, we could not exclude all possible factors that potentially contributed to intravesical recurrence. Since the small sample size may not represent absolute results and limits the ability to conduct subgroup analyses, further large-scale studies are needed to validate our findings.

## Conclusions

The results of this study revealed that dialysis patients did not have inferior surgical outcomes when compared to those with normal renal function after RNU. Dialysis tended to lead to poorer BRFS. However, after adjusting for cancer history, dialysis patients were found to present no difference regarding BRFS. Instead, advanced pathologic tumor stages and previous bladder cancer history were related to the poor BRFS. Therefore, appropriate selection criteria and accurate statistical analyses are mandatory to identify the risk factors for BRFS.

## Acknowledgements

Professor Fu-Wen Liang, for your statistical consultation and assistance.

## Supplementary Material

Figure 2.png

Figure 3.png

Supplementary Figure 1.png

Figure 1.png
