# Supplementary figures and images for: Impact of dialysis on intravesical recurrence and survival outcomes in upper tract urothelial cancer patients undergoing radical nephroureterectomy
Source: Ren Fail. 2025 Feb 7;47(1):2458762. doi: 10.1080/0886022X.2025.2458762 (PMC11809166; doi:10.1080/0886022X.2025.2458762)

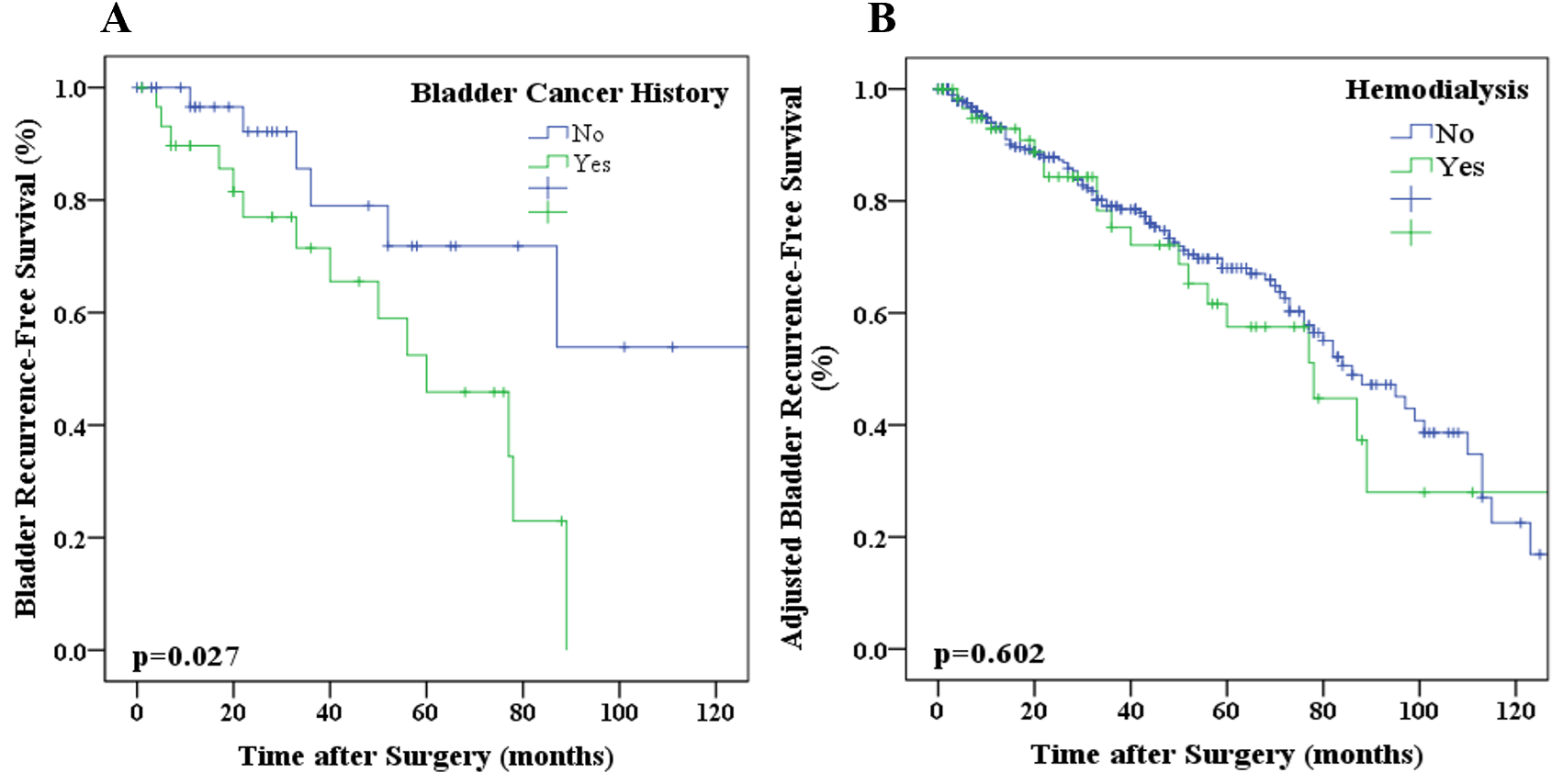

Supplement: Figure 2.png [file IRNF_A_2458762_SM5036.png]

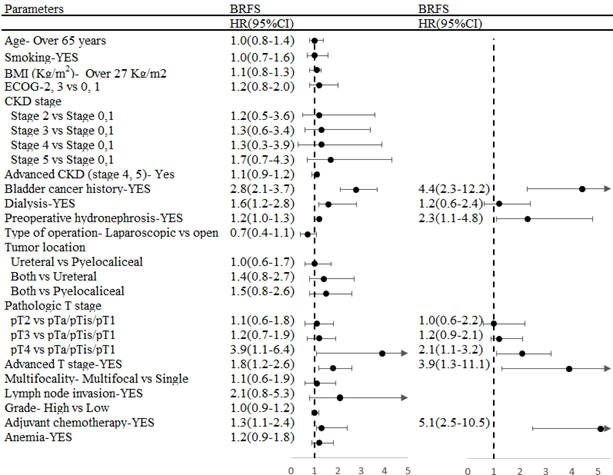

Supplement: Figure 3.png [file IRNF_A_2458762_SM5035.png]

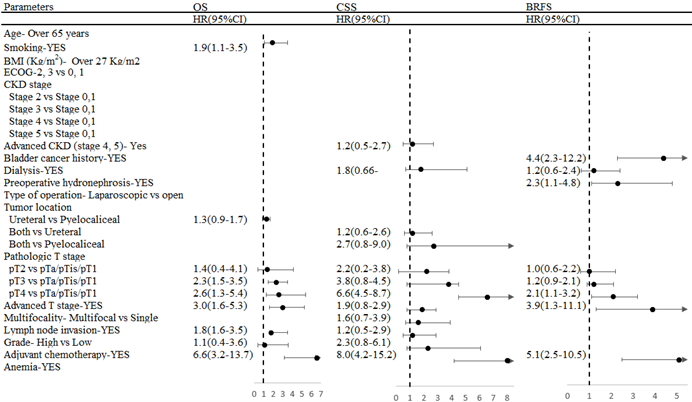

Supplement: Supplementary Figure 1.png [file IRNF_A_2458762_SM5034.png]

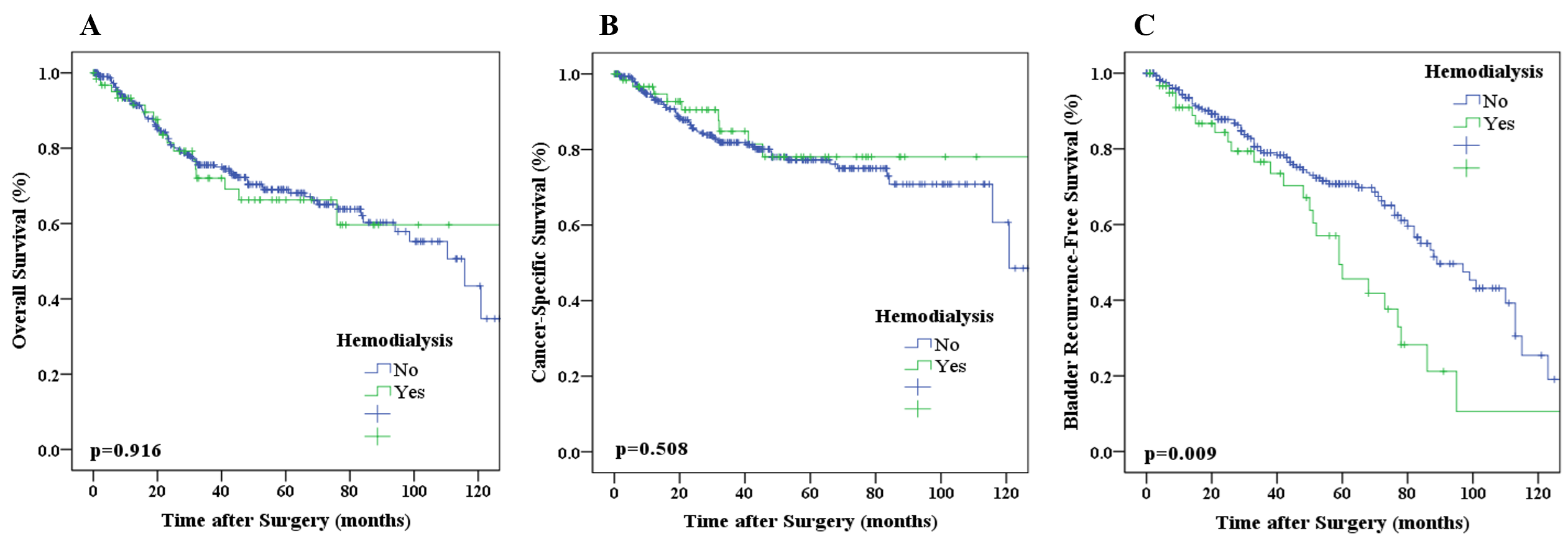

Supplement: Figure 1.png [file IRNF_A_2458762_SM5033.png]
